# Ticagrelor versus prasugrel following PCI in acute coronary syndromes and high-risk coronary artery disease: A meta-analysis of one-year randomized trial outcomes

**DOI:** 10.1016/j.ahjo.2026.100930

**Published:** 2026-10-05

**Authors:** Mohamed Ibrahim Gbreel, Ahmed Samy Badran, Daniel Robinson, Islam Ebeid, Marwa Hassan, Abdelhakim A. Abdelrahman, Jonas Bodanowitz, Stephan Valenta, Christin Valenta, Plamen Kochev, Jasmin Ortak, Hüseyin Ince, Mahmoud Balata

**Affiliations:** a Department of Cardiology, General Organization for Teaching Hospitals and Institutions (GOTHI), Cairo, Egypt; b Faculty of Medicine, Ain Shams University, Cairo, Egypt; c Department of Cardiology, University Hospital of Rostock, Rostock, Germany; d Department of Cardiology, National Institute of Diabetes and Endocrinology, Cairo, Egypt; e Theodor Bilharz Research Institute, Giza, Egypt; f King's College Hospital, Denmark Hill, London, United Kingdom

**Keywords:** Percutaneous coronary intervention, Dual antiplatelet therapy, Ticagrelor, Prasugrel, Acute coronary syndrome, Meta-analysis, P2Y12 inhibitors, Coronary artery disease

## Abstract

**Background:**

Guidelines recommend potent P2Y12 inhibitors over clopidogrel following percutaneous coronary intervention. Whether ticagrelor- or prasugrel-based dual antiplatelet therapy (DAPT) provides superior clinical outcomes following percutaneous coronary intervention (PCI) remains uncertain, particularly across acute coronary syndromes (ACS) and high-risk coronary artery disease (CAD) populations.

**Methods:**

We searched databases for randomized controlled trials comparing ticagrelor- and prasugrel-based DAPT in patients with CAD undergoing PCI with 1-year follow-up. The primary outcomes were major adverse cardiovascular events (MACE) and all-cause mortality. Secondary outcomes included cardiovascular death, myocardial infarction, stroke, stent thrombosis, and major bleeding. Hazard ratios (HRs) with 95% confidence intervals (CIs) were pooled using random-effects models. Sensitivity analyses were performed in ACS populations.

**Results:**

Three trials randomized a total of 7048 patients. In the primary analysis, prasugrel-based therapy was not associated with a statistically significant reduction in MACE compared with ticagrelor-based therapy (HR = 0.83; 95% CI: 0.67–1.03; *p* = 0.09). All-cause mortality (HR = 0.86; 95% CI: 0.68–1.08; *p* = 0.20), cardiovascular death (HR = 0.93; 95% CI: 0.72–1.21; *p* = 0.60), myocardial infarction (HR = 0.80; 95% CI: 0.56–1.14; *p* = 0.22), stroke (HR = 1.04; 95% CI: 0.63–1.73; *p* = 0.87), stent thrombosis (HR = 0.70; 95% CI: 0.45–1.11; *p* = 0.13), and major bleeding (HR = 0.89; 95% CI: 0.68–1.17; *p* = 0.41) were comparable between strategies. ACS-restricted sensitivity analyses demonstrated consistent findings across all outcomes.

**Conclusions:**

Among patients with ACS and high-risk CAD undergoing PCI, current randomized evidence does not establish conclusive superiority between ticagrelor- and prasugrel-based DAPT for 1-year ischemic or bleeding endpoints.

## Graphical abstract

Central Illustration. Ticagrelor Versus Prasugrel Following Percutaneous Coronary Intervention.

This visual abstract displays the primary meta-analysis of one-year clinical outcomes. The forest plot summarizes relative risk estimates across all seven key ischemic and safety endpoints. Points representing hazard ratios lie at the center of their respective 95% confidence intervals.

**Figure:**
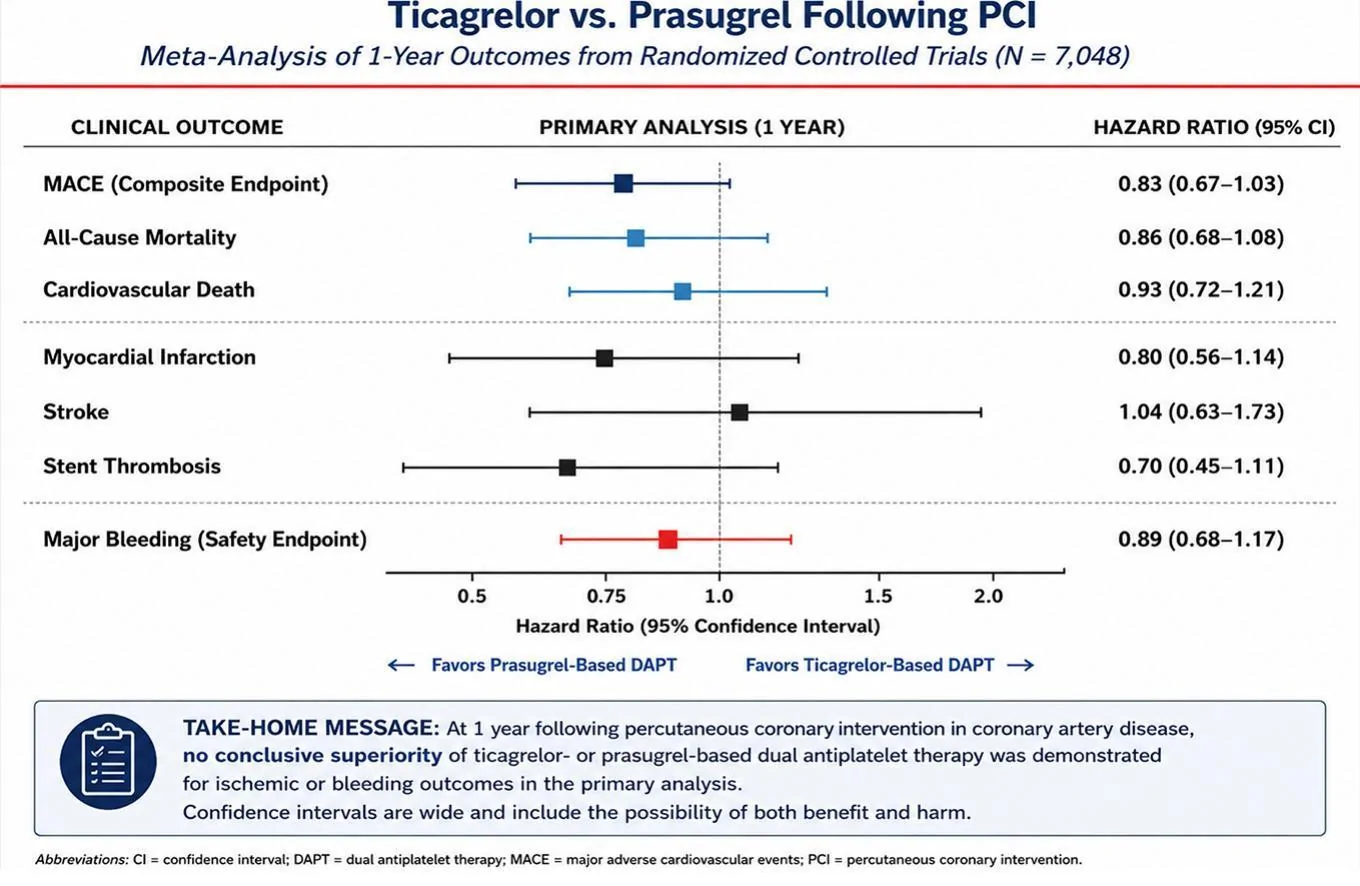

## 1 Introduction

Percutaneous coronary intervention (PCI) remains the cornerstone of treatment for patients with acute coronary syndromes (ACS) and is widely used in chronic coronary artery disease (CAD) [1]. Recurrent ischemic events after PCI remain common despite advances in stent technology and adjunctive pharmacotherapy [2]. Clinical guidelines recommend dual antiplatelet therapy (DAPT) for up to 12 months following PCI in ACS to mitigate local and systemic ischemic events [3].

Ticagrelor and prasugrel provide faster and more potent platelet inhibition than clopidogrel [4]. Current American guidelines endorse both agents equally after PCI, whereas recent European guidelines favor prasugrel in ACS patients undergoing invasive management [5], [6]. The two drugs differ mechanistically. Prasugrel is an irreversible prodrug requiring metabolic activation, whereas ticagrelor is a direct-acting reversible inhibitor with rapid onset and offset [7]. Whether these pharmacologic differences translate into clinically meaningful differences in ischemic or bleeding outcomes remains uncertain. The choice of P2Y12 inhibitor carries considerable economic implications, such as the United States government spending over $750 million on ticagrelor in 2022 alone. Therefore, clarifying the comparative performance of these drugs is essential [8], [9].

Randomized comparisons between ticagrelor and prasugrel have produced conflicting findings. The ISAR-REACT 5 trial reported lower rates of death, myocardial infarction (MI), or stroke with prasugrel in ACS patients undergoing invasive evaluation [10]. In contrast, the PRAGUE-18 trial showed similar ischemic and bleeding outcomes between both agents in patients undergoing primary PCI for MI [11]. More recently, the TUXEDO-2 trial extended this comparison to patients with diabetes mellitus and multivessel coronary artery disease undergoing PCI and again demonstrated no significant difference between treatment strategies [12]. These trials were individually underpowered for several important outcomes.

Individual trials were also constrained by selected population criteria, geographical limits, or premature termination for futility. A synthesis of direct randomized evidence is therefore needed to clarify the comparative efficacy and safety of ticagrelor versus prasugrel in the existing evidence. Therefore, we conducted this systematic review and meta-analysis to compare the one-year clinical efficacy and safety of ticagrelor- versus prasugrel-based DAPT in patients with CAD undergoing PCI.

## 2 Methods

This study followed the recommendations of the Cochrane Handbook for Systematic Reviews of Interventions and was reported according to the Preferred Reporting Items for Systematic Reviews and Meta-Analyses (PRISMA) 2020 statement [13], [14]. The review protocol was registered on PROSPERO (CRD420261353691).

### 2.1 Search strategy and screening

We systematically searched PubMed, Scopus, Web of Science, and the Cochrane Central Register of Controlled Trials through May 1, 2026. The search strategy combined terms related to ticagrelor, prasugrel, PCI, ACS, MI, and CAD. No language restrictions were applied. The full search strategy is provided in Supplementary Table S1.

### 2.2 Eligibility criteria

We defined our inclusion criteria using the PICOS framework. We included RCTs that directly compared ticagrelor- and prasugrel-based DAPT in adult patients with CAD undergoing PCI. Studies enrolling patients with ACS, ST-segment elevation MI (STEMI), or high-risk chronic CAD were eligible. Eligible studies need to report clinical outcomes with at least 1 year of follow-up. Studies evaluating clopidogrel without a direct ticagrelor-versus-prasugrel comparison, observational studies, registry analyses, conference abstracts, reviews, editorials, and duplicate publications were excluded.

### 2.3 Data extraction and endpoints

Two investigators (A.S.B, M.I.G.) independently extracted trial-level data using standardized forms. Extracted variables included study characteristics, enrollment period, sample size, follow-up duration, clinical presentation, procedural characteristics, treatment regimens, and clinical outcomes. Additionally, baseline patient characteristics were also extracted, including age, sex, smoking status, dyslipidemia, diabetes mellitus, hypertension, presentation with STEMI, non–ST-segment elevation MI (NSTEMI), unstable angina, and treatment strategy. Discrepancies were resolved by a third investigator (M.B.). The primary endpoints were all-cause mortality and 1-year major adverse cardiovascular events (MACE), based on individual trial definitions. Secondary endpoints were cardiovascular death, MI, stroke, stent thrombosis, and major bleeding.

### 2.4 Quality assessment

The quality of included studies was independently evaluated by two investigators (M.I.G. and A.S.B.). The Cochrane Risk of Bias 2.0 (RoB 2) tool [15] was used for RCTs. Disagreements were resolved by consensus or by a third author (M.B.).

### 2.5 Statistical analysis

All analyses were performed using RStudio (version 4.4.3, R Foundation for Statistical Computing). Hazard ratios (HRs) with corresponding 95% confidence intervals (CIs) were preferentially extracted for time-to-event outcomes. Pooled effect estimates were calculated using a random-effects model to account for anticipated clinical heterogeneity across PCI populations.

Statistical heterogeneity was assessed using the I^2^ statistic. An I^2^ value >50% was considered indicative of high heterogeneity. Prespecified sensitivity analyses included restriction to ACS populations. We also ran an analysis excluding the PRAGUE 18 trial for sensitivity. Publication bias was not statistically evaluated via Egger's test, as the number of included studies fell below the recommended threshold of 10 [16].

## 3 Results

### 3.1 Study selection and baseline characteristics

Our systematic search identified 5382 records, of which 2441 remained after removing duplicates. Following title and abstract screening, 68 articles underwent full-text assessment, and 3 RCTs met the eligibility criteria for inclusion [10], [11], [12] (Fig. 1).

**Fig. 1:**
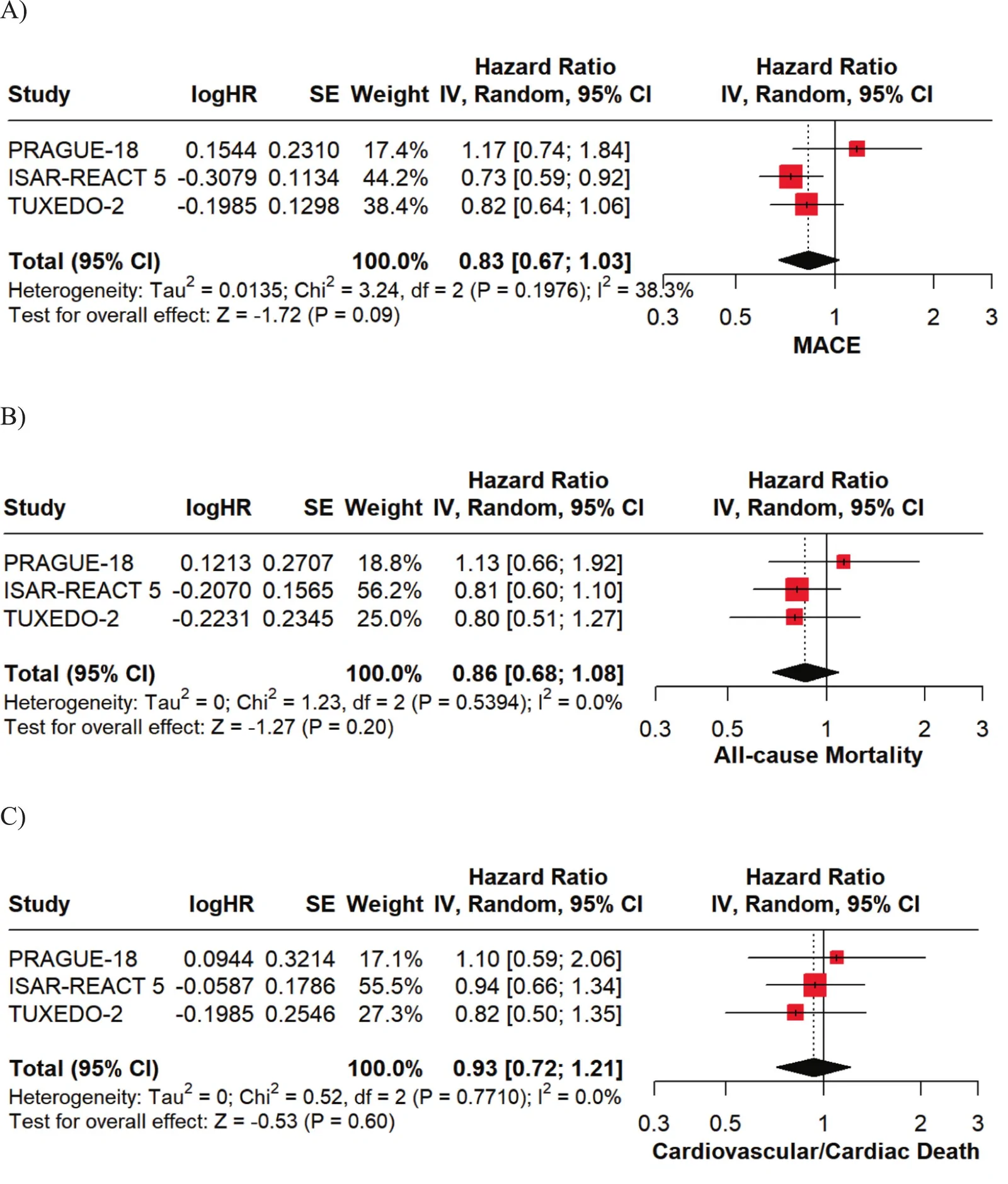
Forest plots of (A) MACE (B) All-cause Mortality (C) Cardiovascular Mortality.

In total, 7048 patients were randomized across the included trials (Table S2). Mean age ranged from 60 to 65 years, and women represented approximately 23% to 29% of the study populations. Both ISAR-REACT 5 and PRAGUE-18 enrolled broad ACS cohorts [10], [11], while TUXEDO-2 restricted enrollment to diabetic patients with multivessel disease [12]. Baseline clinical characteristics and procedural features were generally balanced across treatment groups (Table 1). Trial-level event counts and crude event rates for each outcome, together with the corresponding hazard ratios, are presented in Table S3.

**Table 1:** Patients' baseline characteristics of the included trials.

Trial ID	Arm	Enrolled sample	Age, mean ± SD (years)	Women (%)	Smoker (%)	Dyslipidemia (%)	DM (%)	HTN (%)	STEMI (%)	NSTEMI (%)	Unstable angina (%)	PCI (%)	CABG (%)	Conservative management (%)
TUXEDO-2	Ticagrelor	901	60.48 ± 10.19	29.40%	15.50%	83.10%	100.00%	59.30%	24.4%	34.20%	21.00%	100%	0%	0%
	Prasugrel	899	60.09 ± 10.17	26.60%	16.20%	83.20%	100.00%	61.20%	25.8%	33.30%	19.10%	100%	0%	0%
ISAR-REACT 5	Ticagrelor	2012	64.5 ± 12.0	23.80%	34.10%	58.70%	23.00%	71.30%	41.40%	46.20%	12.40%	83.40%	2.30%	14.20%
	Prasugrel	2006	64.6 ± 12.1	23.80%	33.40%	58.10%	21.40%	69.10%	40.90%	46.10%	13.00%	84.80%	1.80%	13.40%
PRAGUE-18	Ticagrelor	596	61.8 ± 11.2	23.70%	36.90%	38.60%	20.80%	58.70%	95.10%	4.90%	0%	100%	0%	0%
	Prasugrel	634	61.3 ± 11.1	23.40%	38.80%	40.50%	20.00%	55.40%	94.60%	5.40%	0%	100%	0%	0%

CABG = coronary artery bypass grafting; DM = diabetes mellitus; HTN = hypertension; NSTEMI = non–ST-segment elevation myocardial infarction; PCI = percutaneous coronary intervention; SD = standard deviation; STEMI = ST-segment elevation myocardial infarction.

### 3.2 Risk of bias assessment

All three included RCTs were judged as having an overall risk of bias of “some concerns” according to the ROB 2 tool, primarily driven by the open-label design and potential deviations from intended interventions. Randomization, allocation concealment, blinded outcome adjudication, and follow-up completeness remained adequate (Fig. S2).

### 3.3 Primary analysis

#### 3.3.1 Major adverse cardiovascular events and mortality

Prasugrel-based therapy was not associated with a statistically significant reduction in MACE compared with ticagrelor-based therapy (HR: 0.83; 95% CI, 0.67–1.03; *P* = 0.09; I^2^ = 38.3%) (Fig. 1A). All-cause mortality was also similar between groups (HR, 0.86; 95% CI, 0.68–1.08; *P* = 0.20; I^2^ = 0%) (Fig. 1B). Cardiovascular mortality did not differ between treatment strategies (HR, 0.93; 95% CI, 0.72–1.21; *P* = 0.60; I^2^ = 0%) (Fig. 1C).

#### 3.3.2 Ischemic and thrombotic outcomes

MI rates were not significantly different between prasugrel- and ticagrelor-based therapy (HR: 0.80; 95% CI, 0.56–1.14; *P* = 0.22; I^2^ = 49.5%) (Fig. 2A). Stroke also did not differ between treatment strategies (HR: 1.04; 95% CI, 0.63–1.73; *P* = 0.87; I^2^ = 0%) (Fig. 2B). Similarly, stent thrombosis rates were not significantly different (HR: 0.70; 95% CI, 0.45–1.11; *P* = 0.13; I^2^ = 0%) (Fig. 2C).

**Fig. 2:**
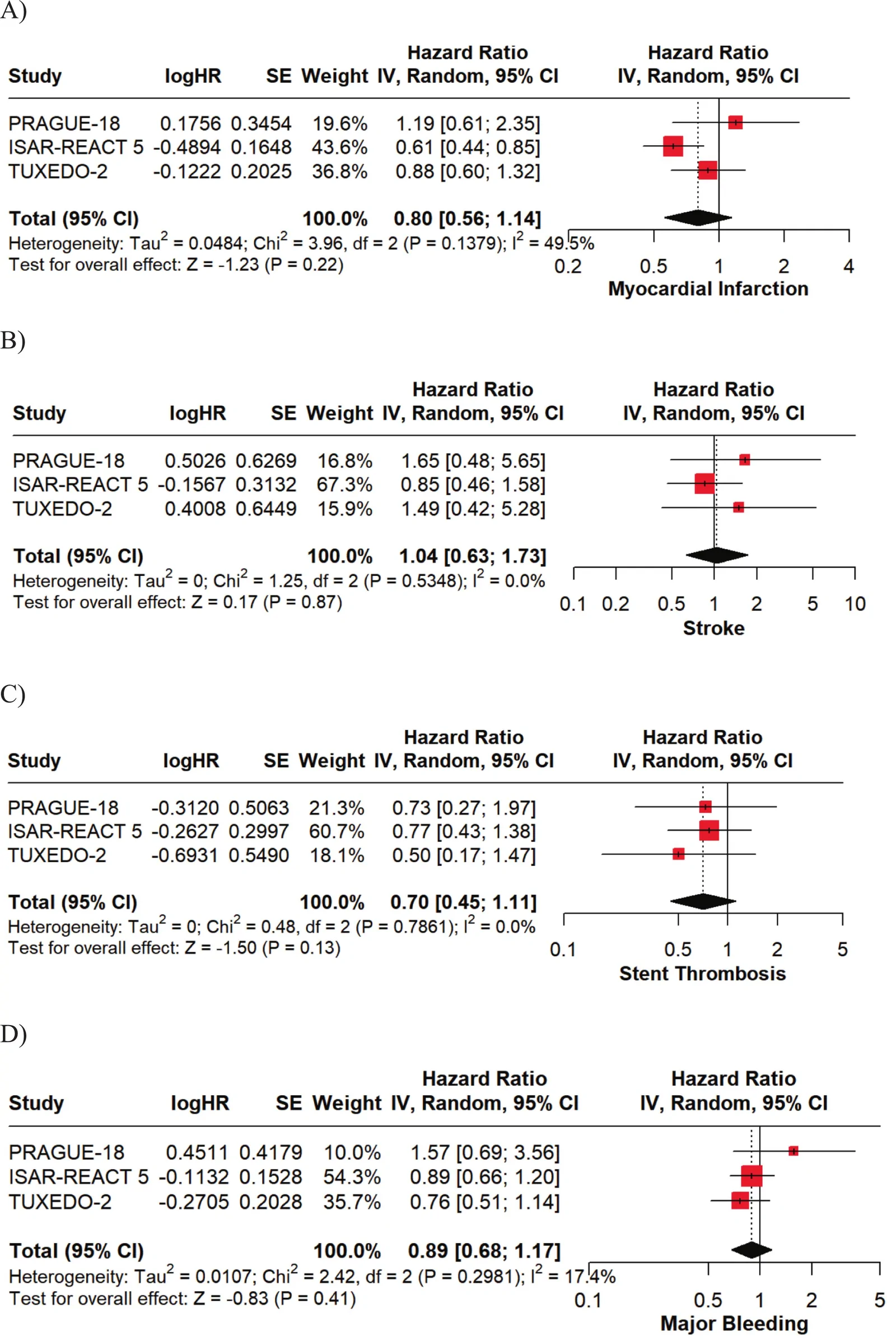
Forest plots of (A) Myocardial Infarction (B) Stroke (C) Stent Thrombosis (D) Major Bleeding.

#### 3.3.3 Bleeding outcomes

Major bleeding rates did not demonstrate a statistically significant difference between groups (HR: 0.89; 95% CI: 0.68–1.17; *p* = 0.41; I^2^ = 17.4%) (Fig. 2D).

#### 3.3.4 Sensitivity analysis restricted to acute coronary syndrome populations

The sensitivity analysis restricted to ACS populations showed no significant difference in MACE (HR: 0.89; 95% CI: 0.57–1.38; *p* = 0.60; I^2^ = 69.0%) (Fig. 3A). All-cause mortality (HR: 0.89; 95% CI: 0.67–1.19; *p* = 0.42; I^2^ = 9.3%) (Fig. 3B) and cardiovascular mortality (HR: 0.98; 95% CI: 0.72–1.33; *p* = 0.88; I^2^ = 0%) (Fig. 3C) did not demonstrate statistically significant differences between groups.

**Fig. 3:**
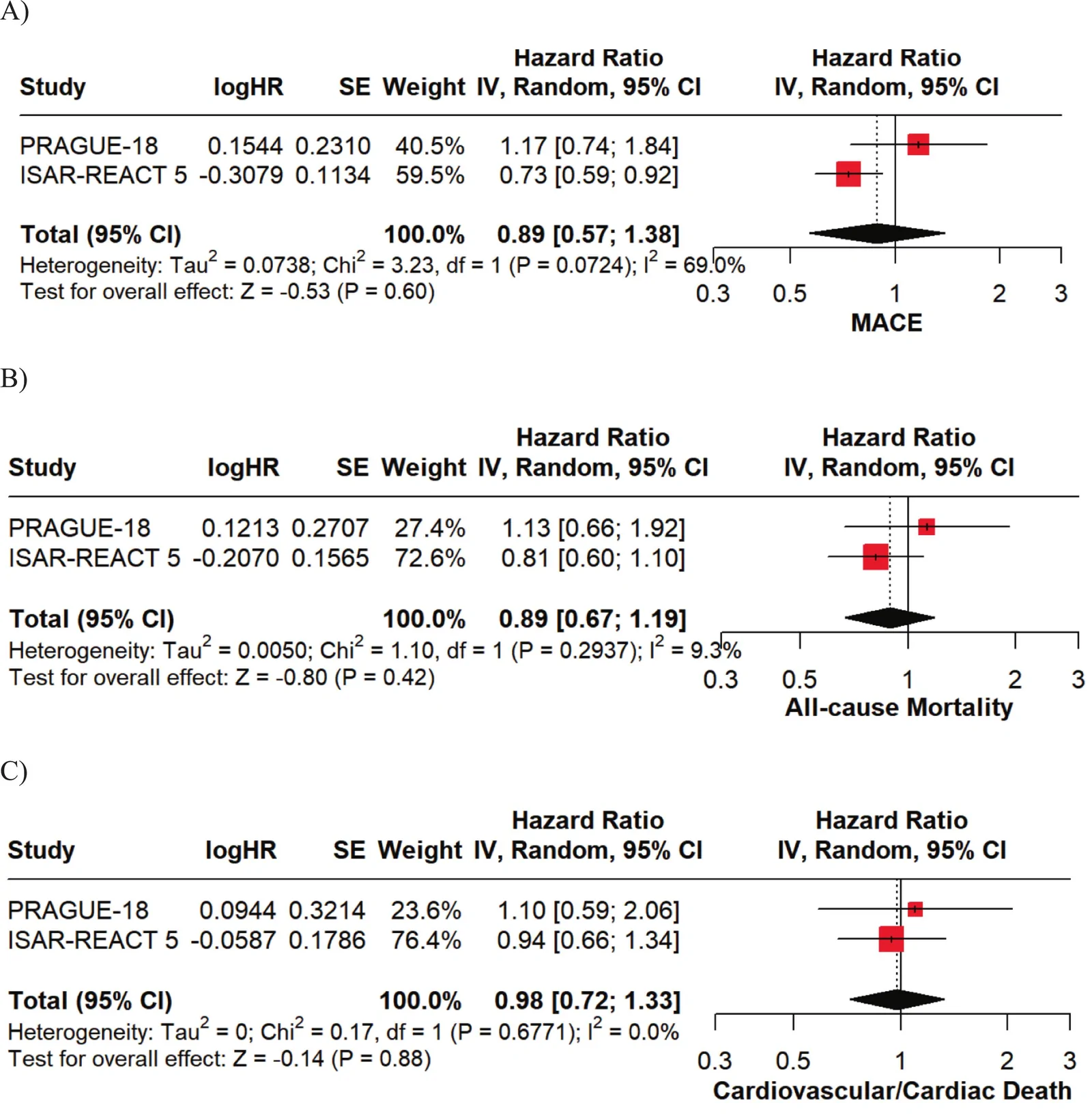
Forest plots of trials including ACS patients (A) MACE (B) All-cause Mortality (C) Cardiovascular Mortality.

Similarly, no significant differences were identified for MI (HR: 0.80; 95% CI: 0.42–1.51; *p* = 0.49; I^2^ = 66.9%) (Fig. 4A), stroke (HR: 0.98; 95% CI: 0.56–1.69; *p* = 0.93; I^2^ = 0%) (Fig. 4B), stent thrombosis (HR: 0.76; 95% CI: 0.46–1.26; *p* = 0.29; I^2^ = 0%) (Fig. 4C), or major bleeding (HR: 1.04; 95% CI: 0.64–1.68; *p* = 0.89; I^2^ = 37.8%) (Fig. 4D).

**Fig. 4:**
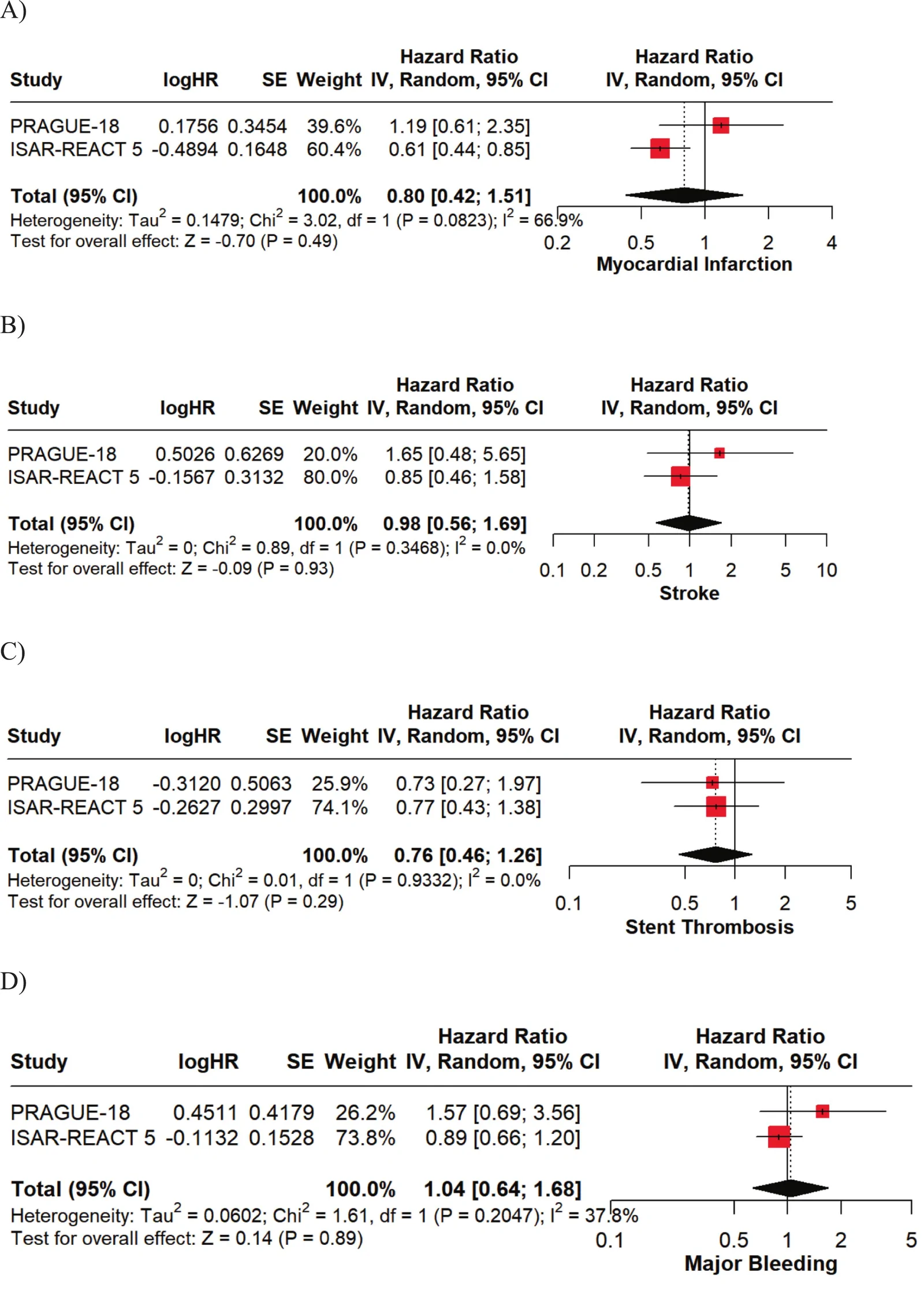
Forest plots of trials including ACS patients (A) Myocardial Infarction (B) Stroke (C) Stent Thrombosis (D) Major Bleeding.

#### 3.3.5 Exploratory sensitivity analysis excluding PRAGUE-18

Given the economic switching bias concerns associated with PRAGUE-18, we performed an additional sensitivity analysis excluding this trial. This analysis showed a significant reduction in MACE favoring the prasugrel-based strategy (HR: 0.77; 95% CI: 0.65–0.91; *p* < 0.01; I^2^ = 0%) (Fig. 5A). All-cause mortality remained statistically comparable between strategies (HR: 0.81; 95% CI: 0.63–1.04; *p* = 0.10; I^2^ = 0%) (Fig. 5B). Rates of cardiovascular death also remained statistically comparable (HR: 0.90; 95% CI: 0.68–1.20; *p* = 0.47; I^2^ = 0%) (Fig. 5C).

**Fig. 5:**
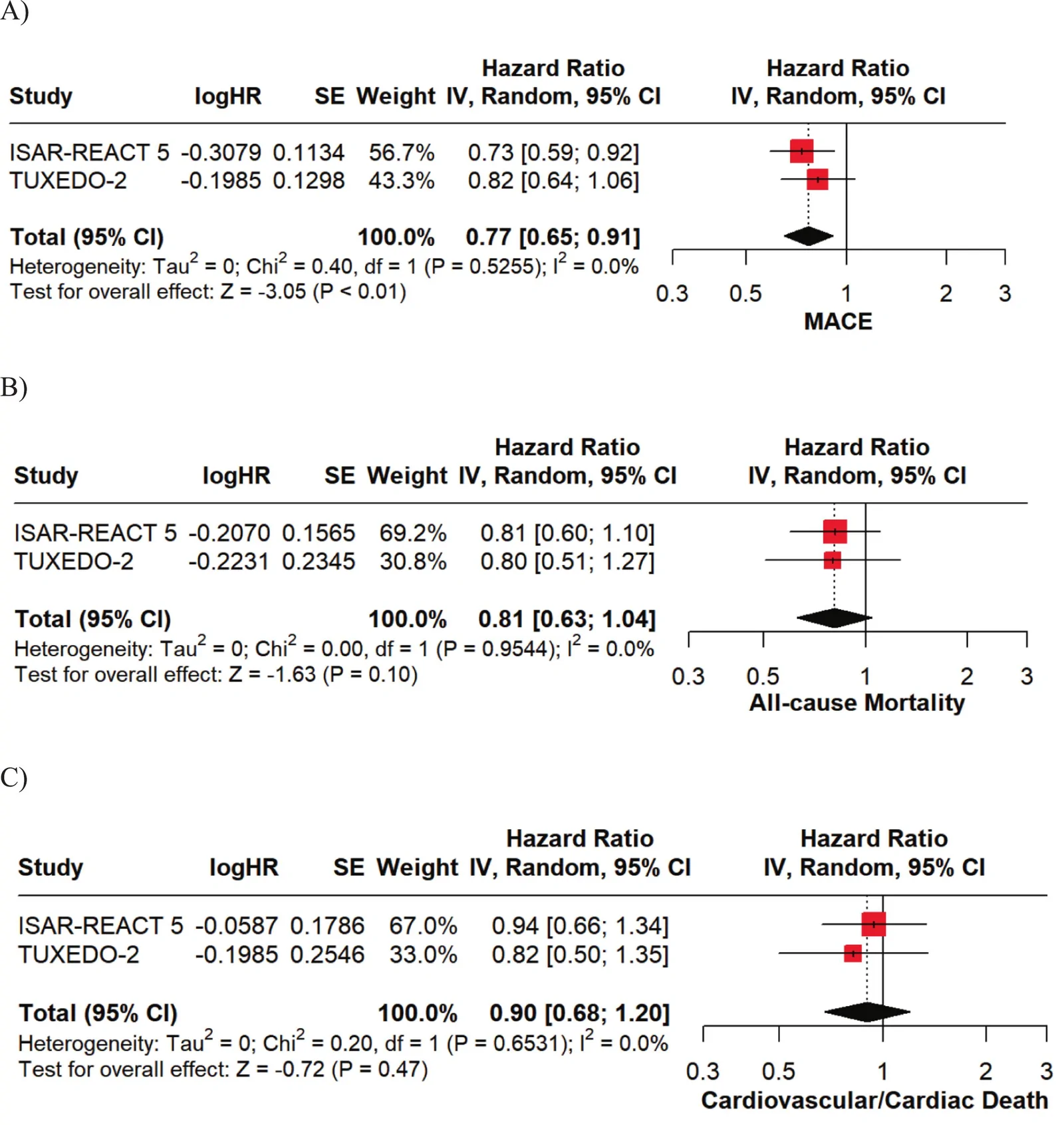
Forest plots of trials excluding PRAGUE-18 (A) MACE (B) All-cause Mortality (C) Cardiovascular Mortality.

Although MI numerically favored prasugrel-based therapy, the difference did not reach statistical significance (HR: 0.72; 95% CI: 0.51–1.03; *p* = 0.08; I^2^ = 49.5%) (Fig. 6A). No statistically significant differences were showed in stroke (HR: 0.95; 95% CI: 0.55–1.65; *p* = 0.86; I^2^ = 0%) (Fig. 6B), stent thrombosis (HR: 0.70; 95% CI: 0.42–1.17; *p* = 0.17; I^2^ = 0%) (Fig. 6C), or major bleeding (HR: 0.84; 95% CI: 0.66–1.07; *p* = 0.16; I^2^ = 0%) (Fig. 6D).

**Fig. 6:**
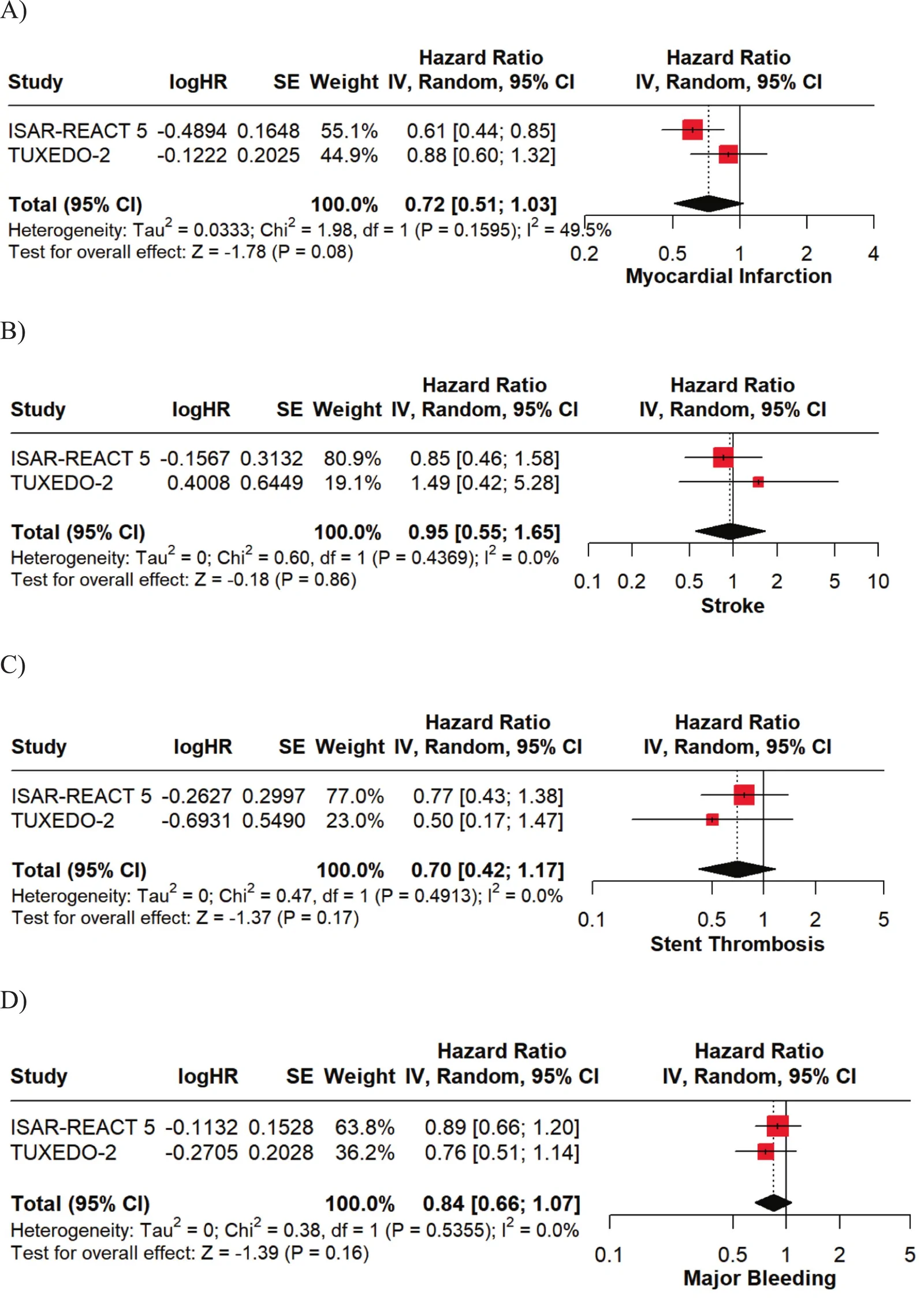
Forest plots of trials excluding PRAGUE-18 (A) Myocardial Infarction (B) Stroke (C) Stent Thrombosis (D) Major Bleeding.

## 4 Discussion

In this meta-analysis of randomized trials including 7048 patients undergoing PCI, the available evidence does not establish superiority of ticagrelor or prasugrel for 1-year ischemic or bleeding outcomes. MACE, all-cause mortality, cardiovascular mortality, MI, stroke, stent thrombosis, and major bleeding were not significantly different between strategies (**Central illustration**). These findings remained consistent in analyses restricted to ACS populations. However, the pooled estimates should be interpreted in the context of substantial clinical and methodological heterogeneity. ISAR-REACT 5 and PRAGUE-18 enrolled ACS populations, whereas TUXEDO-2 enrolled patients with diabetes and multivessel coronary disease.

The direction of effect consistently favored prasugrel across most ischemic endpoints. The signal became statistically significant after exclusion of PRAGUE-18, in which extensive post-discharge switching to clopidogrel may have diluted pharmacologic differences between the study drugs. However, this is an exploratory analysis, and the two-trial model was predominantly driven by the statistical weight of ISAR-REACT 5.

The preference for potent P2Y12 inhibition emerged from landmark comparisons against clopidogrel. TRITON-TIMI 38 showed that prasugrel reduced ischemic events and stent thrombosis in ACS patients undergoing PCI, although with increased major bleeding [17]. PLATO demonstrated reductions in MACE and cardiovascular death with ticagrelor compared with clopidogrel [18]. These trials laid the foundation for prasugrel and ticagrelor in contemporary DAPT and informed guideline recommendations. Neither trial directly compared the two agents, which left uncertainty regarding their relative net clinical benefit. Intensification of antiplatelet therapy also increases bleeding risk, which independently predicts mortality and complications [7]. The optimal P2Y12 inhibitor, therefore, requires a balance between ischemic protection and bleeding risk. The rationale for comparing prasugrel and ticagrelor is biologically important. Both agents provide faster and more potent platelet inhibition than clopidogrel and reduce the impact of high on-treatment platelet reactivity [7]. However, these agents differ mechanistically. Prasugrel is an irreversible thienopyridine prodrug, whereas ticagrelor is a reversible direct-acting inhibitor with rapid onset and offset kinetics [19]. These pharmacologic differences have generated persistent uncertainty regarding comparative efficacy, adherence, and safety in practice.

Our primary analysis did not reproduce a statistically significant reduction in MACE with prasugrel. The consistency of the directional effect across ischemic endpoints and the emergence of significance after exploratory exclusion of PRAGUE-18 suggest that methodological differences between trials may have contributed to the pooled estimate [11]. The PRAGUE-18 trial reported similar early outcomes between prasugrel and ticagrelor, but the interpretation of long-term results was complicated by frequent switching from both agents to clopidogrel because of economic constraints [11]. This may have limited the ability to detect clinically meaningful differences between agents. The exploratory reduction in MACE with prasugrel was directionally consistent with ISAR-REACT 5, and it may suggest the possibility of a more ischemic efficacy with prasugrel in the setting of maintained treatment adherence. Nevertheless, this sensitivity analysis warrants caution as restricting the synthesis to two trials creates an analysis dominated by ISAR-REACT 5. This exploratory finding cannot be construed as independent confirmation of prasugrel superiority, but rather suggests how trial-level adherence patterns and open-label management may influence pooled summary estimates.

Our pooled analysis showed no significant difference in major bleeding between prasugrel and ticagrelor. This finding is clinically relevant because both agents are associated with a higher bleeding risk than clopidogrel [9]. However, the clinical decision between ticagrelor and prasugrel should be viewed within the broader paradigm of bleeding risk stratification. As highlighted by Galli et al. [20], patients identified as High Bleeding Risk (HBR) frequently require tailored antithrombotic regimens rather than uniform 12-month potent DAPT. In HBR cohorts, prolonged potent P2Y12 inhibition may accentuate bleeding hazards without providing ischemic benefit. Modern post-PCI management increasingly incorporates early de-escalation (e.g., switching from potent agents to clopidogrel or dropping aspirin after 1–3 months to maintain potent P2Y12 inhibitor monotherapy) [20]. Therefore, comparative efficacy between ticagrelor and prasugrel represents only one facet of clinical decision-making, which should be integrated with bleeding risk assessment, individualized treatment duration, and targeted de-escalation strategies [7].

The findings of our analysis also parallel emerging observational evidence. A recent TriNetX analysis, including more than 135,000 PCI patients, demonstrated lower rates of MACE, all-cause mortality, MI, and major bleeding with prasugrel compared with ticagrelor at both 1 and 5 years [21]. The consistency of those findings across subgroups and temporal strata reinforces the directional trends observed in our randomized synthesis. Nevertheless, observational comparisons remain vulnerable to residual confounding, treatment-selection bias, and differences in adherence patterns.

Our findings should also be interpreted in the context of ISAR-REACT 5, which remains the pivotal head-to-head randomized comparison between prasugrel and ticagrelor [10]. ISAR-REACT 5 demonstrated a 36% relative reduction in death, MI, or stroke with prasugrel without an increase in major bleeding [10]. These findings influenced the 2023 European guideline recommendations, favoring prasugrel over ticagrelor in ACS patients undergoing PCI [5], [6]. American guidelines recommended both agents similarly [5], [6]. These guideline recommendations may reflect a broader evidence base. Differences in trial design, patient selection, treatment timing, adherence, and bleeding risk contribute to the divergent recommendations between contemporary guidelines. Although our findings do not provide sufficient evidence to modify these recommendations, they instead support continued individualized selection of a potent P2Y12 inhibitor according to ischemic and bleeding risk.

The inclusion of TUXEDO-2 provides additional insight into the interpretation of our findings [12]. This trial enrolled diabetic patients with multivessel coronary artery disease undergoing PCI, a subgroup characterized by increased platelet reactivity and thrombotic risk [22], [23]. Ticagrelor did not achieve noninferiority to prasugrel for the primary composite endpoint and was associated with numerically higher ischemic and bleeding events. Although the study was not powered to establish superiority, it extended the comparison beyond traditional ACS cohorts and added support for at least comparable, and potentially greater, ischemic protection with prasugrel in high-risk PCI populations.

The broader literature surrounding ticagrelor also remains relevant to the interpretation of comparative P2Y12 inhibitor data. Watanabe et al. demonstrated that inclusion of the PLATO trial substantially influenced pooled estimates favoring ticagrelor in network meta-analysis [9]. Exclusion of PLATO attenuated or reversed several efficacy signals, particularly for mortality and MI, while also reducing heterogeneity and inconsistency across analyses [9]. Concerns surrounding geographic variability, endpoint adjudication, and incomplete follow-up within PLATO have therefore continued to influence the interpretation of ticagrelor efficacy [9], [18]. Although our study did not directly compare ticagrelor with clopidogrel, current guideline recommendations supporting ticagrelor were largely derived from PLATO-based evidence.

Adherence may also contribute to differences between prasugrel and ticagrelor in routine clinical practice. Ticagrelor requires twice-daily dosing and is commonly associated with dyspnea, both of which may reduce long-term treatment persistence [7]. Prasugrel uses once-daily dosing and may therefore achieve greater adherence over prolonged follow-up. A pharmacodynamic substudy of ISAR-REACT 5 also suggested stronger platelet inhibition with prasugrel [7], [24]. These factors may partially explain the directional reduction in MI and stent thrombosis observed with prasugrel across observational and randomized datasets, including our own.

### 4.1 Limitations

Several limitations warrant cautious interpretation of our pooled estimates. Only three randomized trials were available, which limited statistical power. All included studies used open-label designs and remained vulnerable to bias related to adherence, crossover, and treatment discontinuation despite blinded endpoint adjudication. The enrolled populations differed with respect to ACS presentation, diabetes burden, and revascularization strategies. The trials also differed in treatment exposure, switching patterns, timing of therapy, and clinical presentations. Given that statistical random-effects models cannot fully adjust for these multifaceted clinical disparities across only three trials, our summary effect estimates should not be viewed as definitive evidence of uniform therapeutic equivalence across all PCI settings. Endpoint definitions also varied across trials, particularly for composite ischemic and bleeding outcomes. Patient-level treatment-effect modifiers could not be evaluated because individual patient data were unavailable. We therefore could not determine whether the relative effects of ticagrelor and prasugrel differ according to age, sex, diabetes, renal dysfunction, adherence patterns, ACS subtype, procedural complexity, prior stroke, body weight, or bleeding risk. These factors may influence the balance between ischemic benefit and bleeding harm and should be addressed in future individual patient data analyses.

## 5 Conclusion

In this meta-analysis evaluating patients with ACS and high-risk CAD undergoing PCI, direct randomized evidence did not demonstrate a statistically significant difference between prasugrel- and ticagrelor-based DAPT in 1-year ischemic or bleeding outcomes. The exploratory reduction in MACE after exclusion of PRAGUE-18 warrants further investigation but does not establish superiority of prasugrel. These findings support continued individualized selection of potent P2Y12 inhibition and warrant additional adequately powered RCTs.

## CRediT authorship contribution statement

**Mohamed Ibrahim Gbreel:** Writing – original draft, Methodology, Formal analysis, Data curation, Conceptualization. **Ahmed Samy Badran:** Writing – original draft, Visualization, Methodology, Formal analysis, Data curation, Conceptualization. **Daniel Robinson:** Writing – original draft, Methodology, Data curation, Conceptualization. **Islam Ebeid:** Writing – original draft, Methodology, Conceptualization. **Marwa Hassan:** Writing – original draft, Methodology, Conceptualization. **Abdelhakim A. Abdelrahman:** Writing – original draft, Methodology, Conceptualization. **Jonas Bodanowitz:** Writing – review & editing, Methodology, Conceptualization. **Stephan Valenta:** Writing – review & editing, Methodology, Conceptualization. **Christin Valenta:** Writing – review & editing, Methodology, Conceptualization. **Plamen Kochev:** Writing – review & editing, Methodology, Conceptualization. **Jasmin Ortak:** Writing – review & editing, Methodology, Conceptualization. **Hüseyin Ince:** Writing – review & editing, Methodology, Conceptualization. **Mahmoud Balata:** Writing – review & editing, Supervision, Project administration, Data curation, Conceptualization.

## Human ethics and consent to participate statement

Because this study did not involve human participants, institutional review board (IRB) approval and informed patient consent were not required.

## Clinical trial number

Not applicable.

## Consent for publication

All authors reviewed and agreed on the final version of the manuscript.

## Funding

No funding received.

## Declaration of competing interest

All authors declared no conflicts of interest.

## Data Availability

All data are available upon request.
