# Supplementary material for: Ticagrelor versus prasugrel following PCI in acute coronary syndromes and high-risk coronary artery disease: A meta-analysis of one-year randomized trial outcomes
Source: Am Heart J Plus. 2026 Oct 5;71:100930. doi: 10.1016/j.ahjo.2026.100930 (PMC13676095; doi:10.1016/j.ahjo.2026.100930)
Supplement: Supplementary file 1 [file mmc1.docx]

**Appendix**

- **Supplementary Tables**
  - **Table S1.** Search strategy for each database.
  - **Table S2.** Summary of the included studies.
  - **Table S3.** Trial-level event counts, event rates, and hazard ratios.
  - **Table S4.** Definitions of endpoints in each trial.
- **Supplementary Figures**
  - **Figure S1.** Flow diagram of study selection.
  - **Figure S2.** Risk of bias of the included studies using the ROB-2 tool.

| **Table S1. Search Strategy for each database.** | | | |
| --- | --- | --- | --- |
| **Databases** | **strategy** | **No. of records** |  |
| **PubMed** | | | |
| #1 | (  "Ticagrelor"[Mesh]  OR ticagrelor[tiab]  OR brilinta[tiab]  OR brilique[tiab]  OR AZD6140[tiab] ) | 5008 |  |
| #2 | (  "Prasugrel Hydrochloride"[Mesh]  OR prasugrel[tiab]  OR effient[tiab]  OR efient[tiab]  OR CS-747[tiab] ) | 3,395 |  |
| #3 | (  "Percutaneous Coronary Intervention"[Mesh]  OR "Coronary Artery Disease"[Mesh]  OR "Acute Coronary Syndrome"[Mesh]  OR "Myocardial Infarction"[Mesh]  OR PCI[tiab]  OR percutaneous coronary intervention[tiab]  OR angioplasty[tiab]  OR coronary stent*[tiab]  OR coronary artery disease[tiab]  OR acute coronary syndrome[tiab]  OR ACS[tiab]  OR STEMI[tiab]  OR NSTEMI[tiab]  OR myocardial infarction[tiab] ) | 541,786 |  |
| #4 | ("Randomized Controlled Trial"[pt] OR "Randomized Controlled Trials as Topic"[MeSH] OR "random*"[tiab] OR "RCT"[tiab] OR "randomised"[tiab] OR "randomized"[tiab] OR "randomly assigned"[tiab] OR "controlled trial*"[tiab]) | 1,990,076 |  |
| #5 | #1 AND #2 AND #3 AND #4 | 501 |  |
| **Cochrane Library** | | | |
| #1 | (  [mh Ticagrelor]  OR ticagrelor:ti,ab,kw  OR brilinta:ti,ab,kw  OR brilique:ti,ab,kw  OR AZD6140:ti,ab,kw ) |  |  |
| #2 | (  [mh "Prasugrel Hydrochloride"]  OR prasugrel:ti,ab,kw  OR effient:ti,ab,kw  OR efient:ti,ab,kw  OR CS-747:ti,ab,kw ) |  |  |
| #3 | (  [mh "Percutaneous Coronary Intervention"]  OR [mh "Coronary Artery Disease"]  OR [mh "Acute Coronary Syndrome"]  OR [mh "Myocardial Infarction"]  OR PCI:ti,ab,kw  OR "percutaneous coronary intervention":ti,ab,kw  OR angioplasty:ti,ab,kw  OR coronary stent*:ti,ab,kw  OR "coronary artery disease":ti,ab,kw  OR "acute coronary syndrome":ti,ab,kw  OR ACS:ti,ab,kw  OR STEMI:ti,ab,kw  OR NSTEMI:ti,ab,kw  OR "myocardial infarction":ti,ab,kw ) |  |  |
| #4 | #1 AND #2 AND #3 | 563 |  |
| **Scopus** | | | |
| #1 | TITLE-ABS-KEY(  (  ticagrelor  OR brilinta  OR brilique  OR AZD6140  ) |  |  |
| #2 | TITLE-ABS-KEY(  prasugrel  OR effient  OR efient  OR CS-747  ) |  |  |
| #3 | TITLE-ABS-KEY(  PCI  OR "percutaneous coronary intervention"  OR angioplasty  OR "coronary stent*"  OR "coronary artery disease"  OR "acute coronary syndrome"  OR ACS  OR STEMI  OR NSTEMI  OR "myocardial infarction"  ) |  |  |
| #4 | TITLE-ABS-KEY(  random* OR trial ) |  |  |
| #5 | #1 AND #2 AND #3 AND #4 | 3,143 |  |
| **Web of Science (WOS)** | |  |  |
| #1 | TS=(  ticagrelor  OR brilinta  OR brilique  OR AZD6140  ) |  |  |
| #2 | TS=(  prasugrel  OR effient  OR efient  OR CS-747  ) |  |  |
| #3 | TS=(  PCI  OR "percutaneous coronary intervention"  OR angioplasty  OR "coronary stent*"  OR "coronary artery disease"  OR "acute coronary syndrome"  OR ACS  OR STEMI  OR NSTEMI  OR "myocardial infarction"  ) |  |  |
| #4 | TS=(  random* OR trial ) |  |  |
| #5 | #1 AND #2 AND #3 AND #4 | 1,175 |  |

| **Table S2. Summary of the included studies** | | | | | | | | |
| --- | --- | --- | --- | --- | --- | --- | --- | --- |
| **Study ID** | **NCT / Registry** | **Design** | **Setting & Countries** | **Study Enrollment Period** | **Total Randomized Sample Size** | **Inclusion criteria** | **Follow-up** | **Primary Outcome** |
| TUXEDO-2 (Bangalore et al., 2026) | CTRI/2019/11/022088 | Investigator-initiated, prospective, open-label, multicenter, 2×2 factorial, randomized trial | 66 clinical sites in India | Feb 2020 – Aug 2024 | 1,800 (Ticagrelor: 901, Prasugrel: 899) | Patients aged ≥18 years with diabetes mellitus and multivessel coronary artery disease (MVD) scheduled for percutaneous coronary intervention (PCI). | 12 months | Composite of death, nonfatal myocardial infarction, stroke, or major bleeding (BARC 3, 4, or 5) at 1 year |
| ISAR-REACT 5 (Schüpke et al., 2019) | NCT01944800 | Investigator-initiated, Phase 4, multicenter, randomized, open-label trial | 23 centers (21 in Germany, 2 in Italy) | Sept 2013 – Feb 2018 | 4,018 (Ticagrelor: 2012, Prasugrel: 2006) | Hospitalized for acute coronary syndrome (STEMI, NSTEMI, or unstable angina) with planned invasive evaluation (coronary angiography). | 12 months | Composite of death, myocardial infarction, or stroke at 1 year |
| PRAGUE-18 (Motovska et al., 2018) | NCT02808767 | Investigator-initiated, Phase 4, multicenter, randomized, open-label, controlled trial | 14 tertiary cardiology centers in the Czech Republic | Ended enrollment May 2016 | 1,230, (Ticagrelor: 596, Prasugrel: 634) | Patients with acute myocardial infarction (STEMI or very high-risk NSTEMI with ongoing ischemia) undergoing a primary PCI (pPCI) strategy. | 12 months | Composite of cardiovascular death, myocardial infarction, or stroke at 1 year |

| Outcome | Trial | Prasugrel, n/N (%) | Ticagrelor, n/N (%) | HR (95% CI) |
| --- | --- | --- | --- | --- |
| MACE | **PRAGUE-18** | **42/634 (6.6)** | **34/596 (5.7)** | **1.167 (0.742–1.835)** |
|  | **ISAR-REACT 5** | **137/2,006 (6.8)** | **184/2,012 (9.1)** | **0.735 (0.588–0.917)** |
|  | **TUXEDO-2** | **107/899 (11.9)** | **129/901 (14.3)** | **0.820 (0.633–1.053)** |
| All-cause death | **PRAGUE-18** | **30/634 (4.7)** | **25/596 (4.2)** | **1.129 (0.664–1.919)** |
|  | **ISAR-REACT 5** | **73/2,006 (3.6)** | **90/2,012 (4.5)** | **0.813 (0.595–1.099)** |
|  | **TUXEDO-2** | **33/899 (3.7)** | **41/901 (4.6)** | **0.800 (0.505–1.266)** |
| Cardiovascular/cardiac death | **PRAGUE-18** | **21/634 (3.3)** | **18/596 (3.0)** | **1.099 (0.585–2.062)** |
|  | **ISAR-REACT 5** | **59/2,006 (2.9)** | **63/2,012 (3.1)** | **0.943 (0.662–1.333)** |
|  | **TUXEDO-2** | **28/899 (3.1)** | **34/901 (3.8)** | **0.820 (0.498–1.351)** |
| Myocardial infarction | **PRAGUE-18** | **19/634 (3.0)** | **15/596 (2.5)** | **1.192 (0.606–2.347)** |
|  | **ISAR-REACT 5** | **60/2,006 (3.0)** | **96/2,012 (4.8)** | **0.613 (0.444–0.847)** |
|  | **TUXEDO-2** | **46/899 (5.1)** | **52/901 (5.8)** | **0.885 (0.595–1.316)** |
| Stroke | **PRAGUE-18** | **7/634 (1.1)** | **4/596 (0.7)** | **1.653 (0.484–5.650)** |
|  | **ISAR-REACT 5** | **19/2,006 (0.9)** | **22/2,012 (1.1)** | **0.855 (0.465–1.587)** |
|  | **TUXEDO-2** | **6/899 (0.7)** | **4/901 (0.4)** | **1.493 (0.420–5.263)** |
| Stent thrombosis | **PRAGUE-18** | **7/634 (1.1)** | **9/596 (1.5)** | **0.732 (0.270–1.965)** |
|  | **ISAR-REACT 5** | **20/2,006 (1.0)** | **26/2,012 (1.3)** | **0.769 (0.429–1.389)** |
|  | **TUXEDO-2** | **5/899 (0.6)** | **10/901 (1.1)** | **0.500 (0.171–1.471)** |
| Major bleeding | **PRAGUE-18** | **15/634 (2.4)** | **9/596 (1.5)** | **1.570 (0.690–3.550)** |
|  | **ISAR-REACT 5** | **80/2,006 (4.0)** | **95/2,012 (4.7)** | **0.893 (0.662–1.205)** |
|  | **TUXEDO-2** | **43/899 (4.8)** | **55/901 (6.1)** | **0.763 (0.513–1.136)** |

**Table S3. Trial-level event counts, event rates, and hazard ratios**

| CI, confidence interval; HR, hazard ratio; MACE, major adverse cardiovascular events.  Event rates are crude proportions calculated as the number of patients experiencing the event divided by the randomized treatment-group total. HRs are the reported time-to-event estimates and were directionally harmonized across studies where necessary.  **Table S4. Definitions of endpoints in each trial.** | | | |
| --- | --- | --- | --- |
| **Trial** | **Major Adverse Cardiovascular Events (MACE)** | **Stent Thrombosis** | **Major Bleeding** |
| **TUXEDO-2** | Composite of death, MI, stroke, or major bleeding | Definite or probable | BARC Type 3, 4, or 5 bleeding |
| **ISAR-REACT 5** | All-cause death, MI, or stroke | Definite or probable | BARC Type 3, 4, or 5 bleeding |
| **PRAGUE-18** | Cardiovascular death, MI, or stroke | Definite | BARC Type 3 or 5 bleeding |
| BARC indicates Bleeding Academic Research Consortium; MI, myocardial infarction. | | | |

**Figure S1.** Flow diagram of study selection.

**Figure S2.** Risk of bias of the included studies using the ROB-2 tool
